# Blending low- and high-intensity cognitive–behavioural therapy in NHS Talking Therapies for anxiety and depression: preliminary evaluation

**DOI:** 10.1192/bjp.2025.10374

**Published:** 2025-09-19

**Authors:** Graham R. Thew, Luke O’Reilly, Alexander Andrews, Dave Brignull, Jade Burton, Krishna Chauhan, Andrew Humphrey, Nevonne Lewis, Charlotte Stride, Caitlyn Teeney, Florence Vaughan-Burleigh, Christina Webb, Martyn Bradshaw, Laurien Broadley, Gabriella Clarke, Natalie Holmes, Edward Rennie, Samantha Sadler, Josef Landsberg, John Pimm, Peggy Postma, Joanne Ryder, Alison Salvadori, David M. Clark

**Affiliations:** Department of Experimental Psychology, University of Oxford, Oxford, UK; NHS Buckinghamshire Talking Therapies, Oxford Health NHS Foundation Trust, High Wycombe, UK; and NHS Oxfordshire Talking Therapies, Oxford Health NHS Foundation Trust, Oxford, UK; NHS Oxfordshire Talking Therapies, Oxford Health NHS Foundation Trust, Oxford, UK; NHS Buckinghamshire Talking Therapies, Oxford Health NHS Foundation Trust, High Wycombe, UK; NHS Hertfordshire Talking Therapies, Hertfordshire Partnership University NHS Foundation Trust, Hatfield, UK; NHS Berkshire Talking Therapies, Berkshire Healthcare NHS Foundation Trust, Bracknell, UK; NHS Oxfordshire Talking Therapies, Oxford Health NHS Foundation Trust, Oxford, UK; NHS Berkshire Talking Therapies, Berkshire Healthcare NHS Foundation Trust, Bracknell, UK; NHS Oxfordshire Talking Therapies, Oxford Health NHS Foundation Trust, Oxford, UK; NHS Berkshire Talking Therapies, Berkshire Healthcare NHS Foundation Trust, Bracknell, UK; NHS Oxfordshire Talking Therapies, Oxford Health NHS Foundation Trust, Oxford, UK; NHS Buckinghamshire Talking Therapies, Oxford Health NHS Foundation Trust, High Wycombe, UK; NHS Hertfordshire Talking Therapies, Hertfordshire Partnership University NHS Foundation Trust, Hatfield, UK; NHS Berkshire Talking Therapies, Berkshire Healthcare NHS Foundation Trust, Bracknell, UK; NHS Oxfordshire Talking Therapies, Oxford Health NHS Foundation Trust, Oxford, UK; NHS Buckinghamshire Talking Therapies, Oxford Health NHS Foundation Trust, High Wycombe, UK; NHS Hertfordshire Talking Therapies, Hertfordshire Partnership University NHS Foundation Trust, Hatfield, UK; NHS Oxfordshire Talking Therapies, Oxford Health NHS Foundation Trust, Oxford, UK; NHS Berkshire Talking Therapies, Berkshire Healthcare NHS Foundation Trust, Bracknell, UK; Department of Experimental Psychology, University of Oxford, Oxford, UK

**Keywords:** Cognitive–behavioural therapy, NHS Talking Therapies for anxiety and depression, mental health outcomes, low-intensity CBT, IAPT

## Abstract

**Background:**

A stepped care approach to treating anxiety and depression is common in mental health services. Low-intensity interventions, typically based on cognitive behavioural principles, are offered first, followed by high-intensity therapy if required. In the English National Health Service Talking Therapies (NHS TT) programme, different types of therapists deliver low- and high-intensity interventions. ‘Stepping up’ therefore involves changing therapist, and often an additional wait, which could both disrupt treatment flow.

In NHS TT, many low-intensity therapists subsequently train at high intensity. Once dual-trained, they typically deliver only high-intensity treatment. With both skillsets, they could theoretically deliver a full stepped care pathway, avoiding potential disruption linked to stepping up.

**Aims:**

To explore a blended treatment approach, where dual-trained therapists move between low- and high-intensity flexibly based on patient need.

**Method:**

Ten dual-trained therapists across 4 services treated 43 patients. Patients with clinical complexities more likely to eventually require high-intensity support were selected. Propensity score matching was used to identify matched control groups from a pool of patients who received stepped care. Treatment characteristics and clinical outcomes were compared. Feedback was obtained from patients, therapists and supervisors.

**Results:**

Compared with matched controls, who received low- then high-intensity treatment, blended treatment required four fewer sessions on average, saving a third of therapist time and was completed 121 days sooner. The reliable recovery rate (54.1%) was 9% higher than the stepped care group (44.7%), which is clinically, although not statistically, significant. Blended treatment showed a non-significantly higher reliable deterioration rate. Patient feedback was positive. Therapists and supervisors highlighted advantages alongside practical challenges.

**Conclusions:**

The blended approach showed promise as an efficient and effective method to deliver therapy when clinicians are dual-trained. Larger-scale studies, and consideration of implementation challenges, are needed. However, results suggest that this approach could potentially offer more flexible and seamless care delivery.

Many mental health services follow a stepped care approach to treating common problems such as anxiety and depression. Typically, this means that people are first offered a ‘low-intensity’ treatment, after which they are stepped up to a more conventional ‘high-intensity’ treatment if required. Low-intensity psychological interventions, based on cognitive–behavioural therapy (CBT) principles, have good empirical support in the treatment of anxiety and depression and are recommended in UK guidelines.^1,2^ These are relatively brief interventions of approximately five to eight sessions, often focusing on specific techniques such as problem solving or thought challenging,^3^ and typically use guided self-help approaches with brief therapist support. Such interventions are therefore relatively efficient to deliver and allow large numbers of people to be treated.

However, not all people benefit from low-intensity treatment and, in these cases, a subsequent course of high-intensity psychological treatment is provided. Evidence-based, high-intensity treatments for anxiety and depression include, among others, CBT, counselling for depression and interpersonal therapy.^1,2^ These are typically offered on an individual basis (in-person or remotely via video, although internet- and group-based treatment is also common), and are longer in duration (e.g. up to 12–20 sessions). The stepped care approach is efficient in that large numbers of people can be seen quickly, and the more resource-intensive, high-intensity interventions can be targeted to those with more treatment-resistant, severe or complex problems.^4^ However, for patients whose difficulties require more than low-intensity intervention alone, this approach can introduce a discontinuity in care. Following the end of their low-intensity intervention, patients may need to transfer to a new clinician, often following a wait period, to undertake high-intensity treatment.

In regard to the National Health Service Talking Therapies for anxiety and depression programme (NHS TT) in England, low-intensity CBT is typically provided by psychological well-being practitioners (PWPs).^4^ PWP training consists of both taught components and in-service experience with qualified PWPs highly skilled in the assessment of anxiety and depression, and in the delivery of a range of low-intensity interventions. To progress their careers, many PWPs go on to undertake high-intensity CBT training. In this training, practitioners learn specific high-intensity treatment protocols and develop skills in using clinical formulation to individualise treatment. Once qualified, most high-intensity CBT therapists return to work in NHS TT services^5^ but typically no longer provide low-intensity interventions, and so have few opportunities to use their low-intensity training and skills directly.

The NHS TT workforce census in 2023 indicated there were 3645 high-intensity CBT therapists and 1331 high-intensity therapists trained in other treatment modalities (full-time equivalents).^6^ In the past 5 years it has been observed that approximately 70–80% of new trainee CBT therapists were previously PWPs, and it is therefore estimated that at least half of the current high-intensity CBT therapist workforce (*n* = 1822) are also trained as PWPs (A. Whittington, personal communication, 2024). This body of dual-trained clinicians could, in principle, deliver the full stepped care approach, thereby avoiding potential disruptions to the therapy process and potentially making treatment more efficient. In Norway’s close equivalent of the NHS TT programme,^7^ the same therapists provide both low- and high intensity therapy.

This service evaluation project aimed to provide a preliminary examination of the impact of dual-trained clinicians offering a blended treatment approach, combining low- and high-intensity CBT flexibly based on clinical need. We measured the clinical outcomes achieved, compared with the standard stepped care pathway, and obtained feedback from patients, therapists and supervisors.

## Method

### Design

This longitudinal observational study examined the clinical outcomes of participants treated using the blended approach. Participants were then compared with matched controls who had received standard care.

### Participants

Forty-three patients were treated using the blended approach between June 2022 and January 2024. Patients were identified based on routine assessments and screening notes. Based on prior examination of previous data-sets from the participating services, we identified six clinical variables associated with poorer recovery rates following low-intensity treatment alone. These were: a duration of the main problem longer than 2 years, re-referral into the service, current mental health comorbidity, current long-term physical health conditions, current interpersonal problems and the presence of other complexity factors (such as childhood trauma, domestic abuse, neurodiversity or recent bereavement). Patients meeting one or more of these criteria were considered suitable for the blended approach. On average, participants met 2.7 of these criteria (range 1–5). Clinical presentations where low-intensity treatment is not empirically supported (post-traumatic stress disorder (PTSD) and social anxiety disorder (SAD)^8^), or where our previous data suggested that high-intensity input was less commonly required (generalised anxiety disorder (GAD)), were not included in the present study.

Of the 43 participants, 25 (58%) were female and 18 (42%) were male. The mean age was 40.81 years (s.d. = 18.42, range 18–82). Regarding ethnicity, 34 (79%) came from White ethnic backgrounds, 2 (5%) from Black backgrounds, 4 (9%) from Asian backgrounds, 2 (5%) from mixed ethnic backgrounds and 1 (2%) from other backgrounds. Long-term physical health conditions were present in 22 (51%) participants. The most common presenting problem was depression (27 participants, 63%), followed by obsessive–compulsive disorder (OCD; 8 participants, 19%), health anxiety (4 participants, 9%), agoraphobia with or without panic (3 participants, 7%) and panic disorder (1 participant, 2%). Two participants were below clinical caseness thresholds for anxiety and depression at baseline, meaning that it was not possible to demonstrate reliable recovery (see Measures, below). These were therefore excluded from the primary analyses.

### Procedure

Individual CBT treatment was provided either in person or via video-conferencing by ten therapists across four NHS TT services, all of whom were dual-trained as PWPs and high-intensity CBT therapists. All received regular supervision from a clinician familiar with the project, which included discussion of the timing and balance of low- and high-intensity components within treatment. The full project team met monthly for additional case discussion and reflection on using the blended approach. Otherwise, treatment was provided following usual practice and service procedures.

The blended approach typically started with the dual-trained therapist conducting an assessment similar to a standard assessment for high-intensity treatment. Such assessments typically explore clinical problems in more depth than routine NHS TT intake assessments. This allowed the therapist to develop an initial formulation that included factors or processes (e.g. rumination) that can sometimes interfere with fully successful implementation of low-intensity techniques. Therapists then implemented the most appropriate treatment components. In the vast majority of cases, this still meant starting with techniques commonly used in low-intensity treatment (e.g. psychoeducation, activity scheduling, thought challenging), before also adding in high-intensity components (e.g. live behavioural experiments, formulating problem cycles, addressing core beliefs) if and when needed. They categorised each treatment session as either low, high or mixed intensity. Mixed-intensity sessions included those where both low- and high-intensity techniques were used, or where high-intensity components were incorporated into the delivery of a low-intensity technique (for example, bringing the patient’s formulation or core beliefs into discussions to tailor how thought challenging is performed). Therapists moved back and forth between low-, mixed- and high-intensity interventions based on their clinical judgement and supervision. Session duration was between 30 and 70 min.

### Measures

We evaluated a range of treatment characteristics and clinical outcomes, all of which are routinely collected in NHS TT services. Treatment characteristics were the number and total duration of sessions, the number of sessions cancelled or not attended and overall treatment duration in days. Clinical outcomes were the NHS TT standard indicators of reliable recovery (primary outcome), reliable improvement and reliable deterioration,^8^ scores on measures of depression (Patient Health Questionnaire 9 (PHQ-9^9^), Generalised Anxiety Disorder 7 (GAD-7^10^) and functional impairment (Work and Social Adjustment Scale (WSAS^11^)), completion of treatment (based on mutual agreement between patient and therapist) and early termination of treatment (earlier than therapist planned).

Reliable improvement or reliable deterioration is demonstrated when the end of treatment scores for depression (PHQ-9), anxiety (GAD-7) or both show a change compared with baseline that is greater than the reliable change index for the measure (4 or more points on GAD-7 and/or 6 or more points on PHQ-9). Reliable recovery is demonstrated when a client starts treatment above the clinical caseness threshold for either anxiety (≥8 on GAD-7) or depression (≥10 on PHQ-9), meets criteria for reliable improvement and, at the end of treatment, shows scores that are below the caseness thresholds for *both* anxiety and depression. Alternative anxiety disorder-specific measures are used in place of the GAD-7 where possible.^8^ Reliable recovery was the prospectively agreed primary outcome, because the English government listed recovery as the target clinical outcome when it announced the creation of NHS TT on World Mental Health Day in 2007,^12^ and the stepped care model used in NHS TT services is explicitly designed to cost-effectively maximise the number of people who recover (see pages 8 and 40 of ref. ^8^). In line with the original business case for NHS TT,^13^ recent research has also shown that the economic impact of treatment is greatest in those who reliably recover.^14^

We collected feedback from patients about their experience of treatment using the NHS TT Patient Experience Questionnaire,^8^ and through discussion with their therapist at the end of treatment. Therapists and supervisors were also invited to complete an online survey eliciting feedback on the blended approach and its potential advantages and disadvantages.

### Analysis

Participants treated using the blended approach were compared with control participants who had received the usual stepped care within the participating services (i.e. low-intensity treatment, followed by high-intensity treatment if required) within the study period. A pool of potential control participants was extracted from routine data within each service. To ensure that the control data-set was comparable regarding treatment format, it included only those who had started treatment with a one-to-one, low-intensity intervention (group- and internet-based low-intensity treatments were excluded). People with a main presenting problem of PTSD, SAD or GAD were excluded, to mirror the blended group.

Propensity score matching^15^ was used to identify two samples of matched controls. Sample 1 selected matches from those who had started treatment at low intensity and went on to receive at least one high-intensity session. This allowed comparison of the blended approach with sequential low-then high-intensity treatment. The control pool sample size was 2934.

Sample 2 selected matches from the entire control pool of those who had started treatment with a one-to-one, low-intensity intervention. This allowed comparison of the blended approach with the overall stepped care approach, which included those who received low-intensity treatment only and those who were later stepped up to high intensity. The control pool sample size was 9540.

Using these two control groups was important because they incorporate different advantages and disadvantages. Sample 1 would be more similar to the blended group because they received both low- and high-intensity sessions, but progressing through a low-intensity treatment and moving to high-intensity means that this group was likely to be more engaged and motivated for treatment. Sample 2 would be more comparable to the blended group in terms of engagement, but would probably have received a greater proportion of low- compared with high-intensity treatment.

Matching was performed in R^16^ version 4.3.1 for MacOS using the MatchIt package.^17^ This allows selection of the most comparable control cases for each treated case based on prespecified baseline variables. For this study, matching was based on the following: the treating service, the primary presenting problem, age, gender, ethnicity, presence of a long-term physical health condition, employment status and baseline scores on PHQ-9, GAD-7 and WSAS. Cases with incomplete data on these variables were excluded. A matching ratio of 5:1 was used, meaning that each treated case was matched with up to 5 control cases; this increases statistical power compared with 1:1 matching. The model used Mahalanobis distance matching within a propensity score calliper; this ensured that only close matches were retained. In sample 1, a calliper of 0.08 was used. Of the 40 patients for whom matches were sought (2 were below caseness at baseline and 1 had incomplete data on the matching variables), 37 were matched successfully to yield a total of 171 control cases. In the larger sample 2, a calliper of 0.05 was used. Of the 40 patients included, 35 were matched successfully to give a total of 171 control cases. Matching quality was assessed by comparing the groups on each matching variable (see Supplementary Tables 1 and 2 available at https://doi.org/10.1192/bjp.2025.10374). Independent *t*-tests and chi-square tests, incorporating weighting to account for the matching ratio, indicated good matching balances with no significant differences on the matching variables.

Comparisons between the blended and control groups were then performed using weighted *t*-tests and chi-square tests, with Cohen’s *d* values and odds ratios calculated as indicators of effect size. To examine the robustness of the findings, sensitivity analyses using a 10:1 matching ratio were performed for both samples, and the same overall pattern of findings was obtained (see Supplementary Tables 3 and 4). Feedback from patients, therapists and supervisors was analysed descriptively. Although the methods and analyses were not preregistered, we followed guidance from an independent consultant with expertise in propensity score matching in order to create the matched control groups prior to the analysis.

## Results

### Treatment balance

**Fig. 1:**
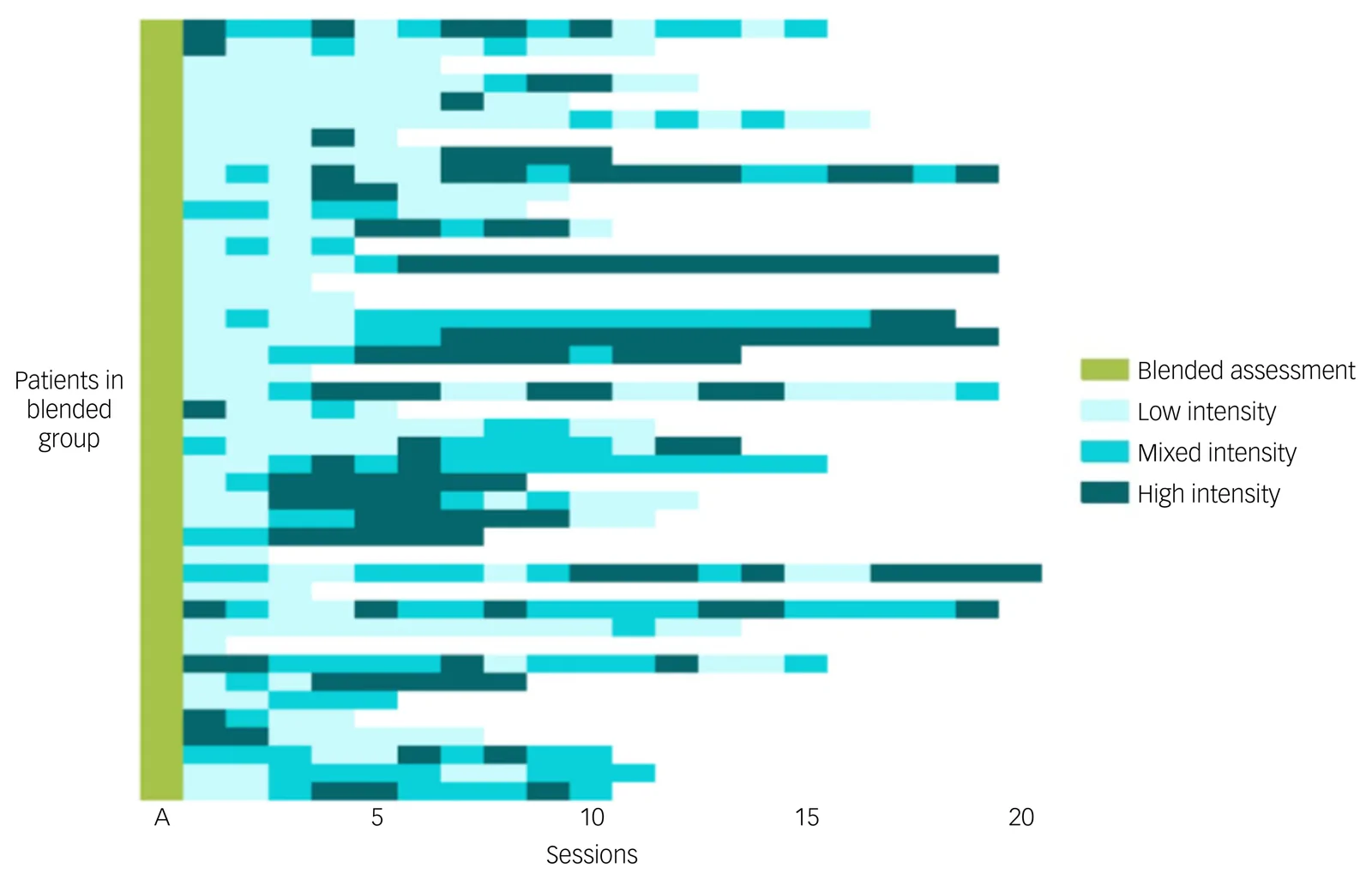
The sequence of low-, mixed- and high-intensity treatment sessions provided for each patient (rows) who received the blended treatment approach. A, assessment session.

The proportion of low-, mixed- and high-intensity sessions for each of the blended group participants is shown in Fig. 1. All participants received at least one low- or mixed-intensity session. On average, the interventions provided were 52.5% low, 21.9% mixed and 25.6% high-intensity.

### Blended versus sequential treatment

Table 1 compares the outcomes of the blended group with the matched controls in sample 1, who received sequential low- then high-intensity treatment. The blended treatment approach was associated with significantly fewer therapy sessions (mean 11.24, s.d. = 0.94) compared with sequential treatment (mean 15.51, s.d. = 0.63), and a significantly shorter total session time (mean difference 289 min). The average duration of blended participant treatment (148 days) was significantly shorter than that for sequential treatment (269 days) and included significantly fewer cancelled or non-attended appointments.

The reliable recovery rate in the blended group (54.1%) was 9% higher than in the sequential group (44.7%), with the odds ratio indicating that patients were 43% more likely to reliably recover in this group compared with controls. Although not statistically significant, meaning that caution in interpretation is warranted, a difference of this magnitude is likely to be considered clinically significant by both practitioners and services. Other findings that were not statistically significant, but may be of clinical interest, were that the blended group showed a higher rate of reliable deterioration and a lower rate of treatment completion compared with controls.

### Blended treatment versus overall stepped care

Table 2 compares the outcomes of the blended group with the matched controls in sample 2, who represent the overall stepped care approach (i.e. low-intensity treatment only, or sequential low-then high-intensity treatment). Participants in the blended group were significantly less likely to drop out of treatment (22.9%) compared with the control group (43.6%), and received a significantly larger dose of treatment in terms of the number of sessions and total session time. The average duration of blended treatment was 149 days, which did not significantly differ from that for the control group (138 days).

The reliable recovery rate in the blended group (54.3%) was 11% higher than in the control group (43.4%), with the odds ratio indicating that patients were 55% more likely to reliably recover in this group compared with controls. As found in sample 1, this difference was not statistically significant although is likely to be considered clinically important.

### Patient experience and feedback

Twenty participants (47%) provided verbal feedback following blended treatment; this comprised 18 treatment completers and 2 participants who were being referred on to another service. Overall, these participants described a positive and helpful treatment experience, with comments highlighting the individual tailoring of treatment. Participants commonly reported that the most helpful aspects of therapy were identifying and challenging thoughts, psychoeducation and having a space to talk and reflect. This aligned with feedback about the main things learned from therapy, which included thought challenging, reflection and self-compassion. Most participants reported being unable to think of anything they found unhelpful, but one reported finding the use of imagery less helpful, one wanted a more counselling-based approach and one felt that the frequency of questionnaires was too high.

Results from the standard NHS TT patient experience questionnaire were also positive, with 14 responses (33%) obtained. All respondents indicated that ‘always’ or ‘most of the time’ they were listened to, felt involved in making treatment choices, were confident in their therapist, were better able to understand and address their difficulties and got the help that mattered to them.

### Therapist and supervisor feedback

Seven therapists (70%) and four supervisors (67%) responded to the survey. When asked about potential advantages, opportunities and positive impacts of the blended approach, respondents highlighted potential benefits for the patient – for example, avoiding interruptions to the patient journey when stepping up to high-intensity treatment. It was also suggested that having high-intensity elements, such as formulation introduced within assessments, may help people understand their difficulties more fully from the start of treatment.

In terms of benefits for therapists, respondents appreciated having greater flexibility in delivering these interventions and how it helped to retain their low-intensity skills. They reported finding it helpful to be able to use their high-intensity knowledge to assist in overcoming barriers during low-intensity work. They also noted some changes to their clinical practice. These included changes to their delivery of low-intensity techniques (such as greater use of Socratic questioning, and offering more space for the patient), and changes to their routine high-intensity work with other patients (such as greater consideration of low-intensity techniques, and earlier consideration of discharge if appropriate). They reported that the blended approach has potential for increases in efficiency – for example, by preventing repetition, avoiding multiple assessments and other potential impacts such as shorter waiting times or fewer sessions.

When asked about potential disadvantages, challenges and negative impacts, respondents highlighted difficulties in becoming familiar with switching between treatment intensities, and raised the possibility that endings may be a challenge when the therapist feels they could offer more treatment. It was also suggested that the inclusion of high-intensity content during the assessment phase may set up expectations of what will be covered in treatment, but that this may be paused in order to start work on low-intensity techniques, which could be confusing for the patient.

Respondents noted practical challenges with implementation of the blended approach, including allowing flexibility within diaries because sessions may be of varying duration, difficulties sourcing suitable cases and finding time for additional supervision. Going forward, therapists suggested that it may be helpful to widen the patient selection criteria – for example, by including those with a duration of their main problem being between 12 and 18 months, and those with a main problem of GAD.

## Discussion

Overall, this study found that, compared with sequential low- then high-intensity treatment (sample 1), the blended approach was associated with significantly fewer sessions, shorter total session time, fewer missed appointments and a total duration of treatment that was on average 121 days shorter. This is likely because the standard stepped care approach typically involves a wait period between finishing low-intensity treatment and starting high-intensity. There is also a change of therapist at this point, which may mean that additional therapy time is needed to allow the patient to feel comfortable with the new therapist, and for the therapist to assess, formulate and plan high-intensity treatment. In contrast, because the blended approach provides a single intervention with one therapist, it may avoid internal delays and ensure continuity of the therapeutic relationship.

On the primary outcome measure, reliable recovery (54.1%) was 9% higher in the blended group. Although not statistically significant, this difference is clinically important and likely to be of interest to services given that the current national NHS TT target for reliable recovery is 48%, moving to 53% over the next 5 years, and that the blended group had more complex presentations than routine referrals. The blended approach may therefore represent an efficient treatment format, requiring fewer resources and obtaining better outcomes compared with sequential interventions.

Rates of reliable improvement showed little difference (64.9% in blended care and 67.3% in sequential care). It is notable that the blended group had slightly higher rates of reliable deterioration compared with the sequential group. Although not statistically significant, further exploration is recommended to clarify whether this finding is representative of the blended approach more widely. The blended group also had a lower rate of treatment completion, which is likely because the sequential group, by definition, had completed a low-intensity intervention and thus may have had a higher average motivation for treatment.

Sample 2 compared the blended approach with the overall stepped care model. Consistent with sample 1, the primary outcome (reliable recovery) was 11% higher in the blended group compared with controls. Results also indicated that the blended approach was associated with significantly less dropout. This suggests that, compared with overall stepped care where most patients have low-intensity treatment only, the blended approach may have been better able to engage and retain this more complex client group. Perhaps because of lower dropout, the blended group received a significantly greater number of sessions and total session time compared with the stepped care controls. Reliable improvement was 65.7% in the blended group and 58.1% in the control group. As in sample 1, reliable deterioration was higher in the blended group (14.3%) compared with controls (10.6%). The total duration of treatment was not significantly different between groups, which also highlights the potential efficiency of the blended approach. It should be noted that the statistical significance observed for dropout was not present in the sensitivity analysis. Replication is therefore needed, but the observed magnitude of the difference in dropout rates in both analyses (14–21%) would be of clinical importance to services.

Feedback from the blended group participants was highly positive, although because they were not familiar with how the blended approach differs from standard care, they could not comment on this specifically. Some feedback was given directly to the therapist, and it was not possible to obtain feedback from those who dropped out, meaning that response and sampling biases may be present. Future studies could seek to understand individuals’ experiences of the blended approach in more depth.

This therapist and supervisor survey highlighted several potential benefits for patients, therapists and services, in particular an ability to overcome the discontinuity in care that results from the transition from low- to high-intensity treatment. Feedback suggested that therapists enjoyed working in this way, using a broader range of skills more flexibly. This indicates the possibility that incorporating blended work into a therapist’s caseload might serve to increase variety within their role and potentially support job satisfaction and well-being, although further studies are required to evaluate this.

However, therapists and supervisors also raised several challenges posed by this approach, particularly issues around operational implementation – for example, organising therapists’ diaries and workplans when session lengths may vary at different stages of treatment. Services considering the blended approach would need to review how to address such challenges; for example, in this study each therapist provided blended treatment to one or two cases at a time within their usual caseload, but other services may find it more feasible to have a small number of therapists specialising in blended work.

In regard to supervision, therapists also reflected on whether some patients may benefit more from this approach than others. The present sample included neurodiverse patients and older adults, for which therapists felt that keeping the same clinician was particularly valuable. However, they raised the question of whether some patients may prefer a clearer distinction between treatment intensities, or specifically benefit from a period of consolidation in between. To our knowledge, these questions have not been addressed in the literature. Larger-scale investigations of the step-up process would help more clearly define the subgroup of patients most likely to benefit from a blended approach.

Strengths of this study include the involvement of multiple services, close treatment supervision and the use of methods to ensure high-quality matches: i.e. a large control pool, multiple matching variables, a one-to-many matching ratio, quality checks and sensitivity analyses. The main limitation is that participants were not randomised to blended treatment or usual care, which would have been the strongest design but was not possible to implement. However, propensity score matching is a good alternative in that a highly comparable group of control cases can be selected from a large pool. Sample size was pragmatically determined by the availability and clinical capacity of the therapists. It is possible that some analyses may have been underpowered to detect smaller effects, although the estimated effect sizes provide an indication where this may be the case. Lastly, it was not possible to obtain patient experience data for the control pool participants, meaning that we could not compare patient feedback directly across groups.

Overall, the findings suggest that, where dual-trained clinicians are available, the blended approach shows potential as an efficient and effective method to deliver therapy to those patients who may eventually require some high-intensity intervention in addition to low-intensity intervention. There are operational challenges to implementation that would need to be considered by services. Further research with larger samples is warranted to further explore the feasibility and clinical and cost-effectiveness of this approach. Health economic evaluation would need to consider the delivery costs of using a high-intensity therapist to deliver the full intervention. We estimate that these costs would be comparable to those in the sample 1 control group, but probably higher than those in the sample 2 control group, who had a greater proportion of low- intensity sessions. Randomisation of participants to blended care or usual care would be the strongest approach. The costs of both approaches would need to be examined in relation to the subsequent clinical and economic benefits observed. Existing evidence suggests that economic benefits are particularly large when individuals reliably recover.^14^

Further work to understand patients’ experiences of blended versus sequential approaches, clarifying how to select those patients most likely to benefit and investigating potential negative effects, would be beneficial. If this approach becomes common, it would also be helpful to develop suitable measures to assess therapist competence delivering this approach. The blended approach has potential to provide more flexible and seamless delivery of care and, if supported in further studies, may be of interest to existing services where stepped care models are used, and to those developing new services.

## Supplementary Material

The supplementary material is available online at https://doi.org/10.1192/bjp.2025.10374

Supplementary Tables

## Figures and Tables

**Table 1:** Comparison of clinical outcomes using the blended approach versus sequential low-intensity then high-intensity treatment (sample 1)

Outcome variable	Blended group, % (n = 37)	Matched controls, % (*n* = 171)	Odds ratio [95% CI]	Test statistic
Reliable recovery	54.1	44.7	1.43 [0.82, 2.51]	*χ*^2^(1) = 1.83, *P* = 0.310
Reliable improvement	64.9	67.3	0.91 [0.51, 1.65]	*χ*^2^(1) = 0.14, *P* = 0.777
Reliable deterioration	13.5	6.5	2.50 [0.95, 7.47]	*χ*^2^(1) = 2.85, *P* = 0.145
Completed treatment	67.6	75.4	0.71 [0.38, 1.32]	*χ*^2^(1) = 1.57, *P* = 0.327
Early termination (dropout)	24.3	21.4	1.19 [0.61, 2.33]	*χ*^2^(1) = 0.26, *P* = 0.693
	**Mean (s.e.)**	**Mean (s.e.)**	**Cohen’s *d***	
Number of sessions	11.24 (0.94)	15.51 (0.63)	0.55	*t*(206) = –3.76, *P* < 0.001
Total session time (min)	518.24 (45.17)	807.41 (36.47)	0.65	*t*(206) = –4.98, *P* < 0.001
Number of cancelled or non-attended sessions	1.59 (0.33)	2.61 (0.23)	0.36	*t*(206) = –2.55, *P* = 0.012
Total duration of treatment (days)	147.59 (15.20)	269.11 (14.11)	0.71	*t*(206) = –5.86, *P* < 0.001
PHQ-9 change	6.70 (1.14)	6.40 (0.53)	0.04	*t*(206) = 0.24, *P* = 0.809
GAD-7 change	5.95 (0.99)	5.05 (0.43)	0.16	*t*(206) = 0.83, *P* = 0.405
WSAS change	6.32 (1.56)	6.48 (0.83)	0.01	*t*(202) = –0.09, *P* = 0.930

PHQ-9, Patient Health Questionnaire 9; GAD-7, Generalised Anxiety Disorder 7; WSAS, Work and Social Adjustment Scale. All descriptives and statistical tests incorporated weighting to account for the matching ratio. Cohen’s *d* was calculated using the pooled standard deviation.

**Table 2:** Comparison of clinical outcomes using the blended approach versus overall stepped care treatment (sample 2)

Outcome variable	Blended group, % (n = 35)	Matched controls, % (*n* = 171)	Odds ratio [95% CI]	Test statistic
Reliable recovery	54.3	43.4	1.55 [0.89, 2.73]	*χ*^2^(1) = 2.43, *P* = 0.243
Reliable improvement	65.7	58.1	1.40 [0.79, 2.50]	*χ*^2^(1) = 1.25, *P* = 0.409
Reliable deterioration	14.3	10.6	1.31 [0.56, 3.14]	*χ*^2^(1) = 0.65, *P* = 0.534
Completed treatment	68.6	51.3	2.13 [1.20, 3.82]	*χ*^2^(1) = 6.41, *P* = 0.065
Early termination (dropout)	22.9	43.6	0.38 [0.20, 0.70]	*χ*^2^(1) = 9.96, *P* = 0.025
	**Mean (s.e.)**	**Mean (s.e.)**	**Cohen’s *d***	
Number of sessions	11.34 (0.95)	8.28 (0.59)	0.41	*t*(204) = 2.74, *P* = 0.007
Total session time (min)	521.71 (45.75)	381.39 (36.10)	0.32	*t*(204) = 2.41, *P* = 0.017
Number of cancelled or non-attended sessions	1.66 (0.35)	2.10 (0.16)	0.21	*t*(204) = –1.17, *P* = 0.242
Total duration of treatment (days)	149.43 (15.40)	137.63 (11.15)	0.09	*t*(204) = 0.62, *P* = 0.536
PHQ-9 change	6.83 (1.15)	5.94 (0.50)	0.14	*t*(203) = 0.71, *P* = 0.478
GAD-7 change	5.86 (1.01)	4.71 (0.43)	0.20	*t*(204) = 1.05, *P* = 0.296
WSAS change	6.14 (1.58)	5.44 (0.72)	0.08	*t*(196) = 0.41, *P* = 0.682

PHQ-9, Patient Health Questionnaire 9; GAD-7, Generalised Anxiety Disorder 7; WSAS, Work and Social Adjustment Scale. All descriptives and statistical tests incorporated weighting to account for the matching ratio. Cohen’s *d* calculated using the pooled standard deviation.

## Data Availability

The data that support the findings of this study are available on request from the corresponding author. The data are not publicly available due to privacy and ethical restrictions.
