# Supplementary material for: Blending low- and high-intensity cognitive–behavioural therapy in NHS Talking Therapies for anxiety and depression: preliminary evaluation
Source: Br J Psychiatry. Author manuscript; Available in PMC 2026 Jan 29. (PMC7618677; doi:10.1192/bjp.2025.10374)
Supplement: Supplementary Tables [file EMS212210-supplement-Supplementary_Tables.docx]

**Supplementary Materials**

**Blending low-intensity and high-intensity cognitive behavioural therapy in NHS Talking Therapies for anxiety and depression: A preliminary evaluation**

Table S1. Assessment of matching quality: Comparison of the blended and control groups (Sample one) on the matching variables within the matched dataset.

| Baseline Variable | Blended Group  (N = 37) | Matched Controls  (N = 171) | Test Statistic |
| --- | --- | --- | --- |
| PHQ-9 Mean (SE) | 15.65 (1.04) | 15.45 (0.41) | *t*(206) = 0.18, *p* = .861 |
| GAD-7 Mean (SE) | 13.24 (0.74) | 12.99 (0.33) | *t*(206) = 0.31, *p* = .755 |
| WSAS Mean (SE) | 19.76 (1.51) | 20.58 (0.66) | *t*(206) = -0.50, *p* = .620 |
| Age Mean (SE) | 40.41 (3.02) | 40.06 (1.20) | *t*(206) = 0.11, *p* = .915 |
| Gender |  |  | χ^2^ (1) = 0.15, *p* = .775 |
| % Female | 59.5 | 56.8 |  |
| % Male | 40.5 | 43.2 |  |
| % Non-Binary | 0 | 0 |  |
| % Other | 0 | 0 |  |
| Ethnicity |  |  | χ^2^ (4) = 6.65, *p* = .397 |
| % White | 81.1 | 87.7 |  |
| % Black | 2.7 | 5.3 |  |
| % Mixed | 5.4 | 1.6 |  |
| % Asian | 8.1 | 2.3 |  |
| % Other | 2.7 | 3.1 |  |
| Service |  |  | χ^2^ (3) = 1.09, *p* = .884 |
| % Berkshire | 29.7 | 26.1 |  |
| % Buckinghamshire | 5.4 | 3.2 |  |
| % Hertfordshire | 32.4 | 36.3 |  |
| % Oxfordshire | 32.4 | 34.4 |  |
| Presenting Problem |  |  | χ^2^ (4) = 1.29, *p* = .957 |
| % Depression | 64.9 | 66.0 |  |
| % Agoraphobia | 2.7 | 3.4 |  |
| % Panic Disorder | 5.4 | 7.3 |  |
| % OCD | 18.9 | 13.9 |  |
| % Health Anxiety | 8.1 | 9.4 |  |
| % Other | 0 | 0 |  |
| Long-term physical health condition |  |  | χ^2^ (1) < 0.01, *p* = .984 |
| % No | 54.1 | 53.9 |  |
| % Yes | 45.9 | 46.1 |  |
| Employment status |  |  | χ^2^ (2) = 2.84, *p* = .452 |
| % Employed/Student | 83.8 | 86.1 |  |
| % Unemployed | 5.4 | 8.6 |  |
| % Long term sickness | 0 | 0 |  |
| % Homemaker | 0 | 0 |  |
| % Volunteer | 0 | 0 |  |
| % Retired | 10.8 | 5.2 |  |

*Notes.* All tests applied weighting to account for the matching ratio. Empty categories were removed prior to Chi-square testing where relevant.

Table S2. Assessment of matching quality: Comparison of the blended and control groups (Sample two) on the matching variables within the matched dataset.

| Baseline Variable | Blended Group  (N = 35) | Matched Controls  (N = 171) | Test Statistic |
| --- | --- | --- | --- |
| PHQ-9 Mean (SE) | 15.66 (1.09) | 15.34 (0.48) | *t*(204) = 0.27, *p* = .790 |
| GAD-7 Mean (SE) | 13.09 (0.77) | 13.05 (0.35) | *t*(204) = 0.04, *p* = .967 |
| WSAS Mean (SE) | 19.63 (1.60) | 19.54 (0.63) | *t*(204) = 0.05, *p* = .959 |
| Age Mean (SE) | 41.14 (3.15) | 39.89 (1.29) | *t*(204) = 0.37, *p* = .714 |
| Gender |  |  | χ^2^ (1) = 0.62, *p* = .558 |
| % Female | 60.0 | 54.6 |  |
| % Male | 40.0 | 45.4 |  |
| % Non-Binary | 0 | 0 |  |
| % Other | 0 | 0 |  |
| Ethnicity |  |  | χ^2^ (4) = 2.52, *p* = .800 |
| % White | 82.9 | 85.7 |  |
| % Black | 2.9 | 0.6 |  |
| % Mixed | 5.7 | 4.1 |  |
| % Asian | 5.7 | 7.9 |  |
| % Other | 2.9 | 1.7 |  |
| Service |  |  | χ^2^ (3) = 0.83, *p* = .922 |
| % Berkshire | 25.7 | 24.7 |  |
| % Buckinghamshire | 5.7 | 3.4 |  |
| % Hertfordshire | 34.3 | 38.1 |  |
| % Oxfordshire | 34.3 | 33.7 |  |
| Presenting Problem |  |  | χ^2^ (4) = 1.65, *p* = .914 |
| % Depression | 68.6 | 67.4 |  |
| % Agoraphobia | 2.9 | 0.7 |  |
| % Panic Disorder | 5.7 | 6.3 |  |
| % OCD | 17.1 | 18.0 |  |
| % Health Anxiety | 5.7 | 7.6 |  |
| % Other | 0 | 0 |  |
| Long-term physical health condition |  |  | χ^2^ (1) = 0.29, *p* = .685 |
| % No | 57.1 | 60.9 |  |
| % Yes | 42.9 | 39.1 |  |
| Employment status |  |  | χ^2^ (2) = 0.01, *p* = .997 |
| % Employed/Student | 82.9 | 83.3 |  |
| % Unemployed | 5.7 | 5.7 |  |
| % Long term sickness | 0 | 0 |  |
| % Homemaker | 0 | 0 |  |
| % Volunteer | 0 | 0 |  |
| % Retired | 11.4 | 11.0 |  |

*Notes.* All tests applied weighting to account for the matching ratio. Empty categories were removed prior to Chi-square testing where relevant.

Table S3. Results of Sensitivity Analysis using a 10:1 matching ratio (Sample one).

| Outcome Variable | Blended Group  %  (N = 37) | Matched Controls  %  (N = 326) | Odds Ratio  [95%CI] | Test Statistic |
| --- | --- | --- | --- | --- |
| Reliable Recovery | 54.1 | 43.0 | 1.55 [0.89, 2.73] | χ^2^ (1) = 4.46, *p* = .213 |
| Reliable Improvement | 64.9 | 63.4 | 1.09 [0.61, 1.95] | χ^2^ (1) = 0.08, *p* = .866 |
| Reliable Deterioration | 13.5 | 7.2 | 2.13 [0.84, 5.95] | χ^2^ (1) = 3.89, *p* = .177 |
| Completed treatment | 67.6 | 73.0 | 0.79 [0.42, 1.45] | χ^2^ (1) = 1.30, *p* = .486 |
| Early termination (dropout) | 24.3 | 22.6 | 1.06 [0.55, 2.05] | χ^2^ (1) = 0.15, *p* = .816 |
|  | Mean (SE) | Mean (SE) | Cohen’s *d* |  |
| Number of sessions | 11.24 (0.94) | 14.90 (0.46) | 0.46 | *t*(361) = -3.49, *p* < .001 |
| Total session time (mins) | 518.24 (45.13) | 772.96 (26.86) | 0.54 | *t*(361) = -4.85, *p* < .001 |
| Number of cancelled or not attended sessions | 1.59 (0.33) | 2.62 (0.16) | 0.36 | *t*(361) = -2.79, *p* = .006 |
| Total duration of treatment (days) | 147.59 (15.18) | 256.63 (11.12) | 0.57 | *t*(361) = -5.79, *p* < .001 |
| PHQ-9 change | 6.70 (1.15) | 6.01 (0.36) | 0.11 | *t*(360) = 0.57, *p* = .569 |
| GAD-7 change | 5.95 (0.99) | 5.00 (0.34) | 0.16 | *t*(361) = 0.91, *p* = .365 |
| WSAS change | 6.32 (1.56) | 5.60 (0.57) | 0.07 | *t*(355) = 0.43, *p* = .669 |

*Notes.* All descriptives and statistical tests incorporated weighting to account for the matching ratio. Cohen’s *d* calculated using the pooled standard deviation.

Table S4. Results of Sensitivity Analysis using a 10:1 matching ratio (Sample two).

| Outcome Variable | Blended Group  %  (N = 35) | Matched Controls  %  (N = 334) | Odds Ratio  [95%CI] | Test Statistic |
| --- | --- | --- | --- | --- |
| Reliable Recovery | 54.3 | 47.2 | 1.32 [0.76, 2.31] | χ^2^ (1) = 1.85, *p* = .429 |
| Reliable Improvement | 65.7 | 63.1 | 1.14 [0.66, 2.04] | χ^2^ (1) = 0.27, *p* = .766 |
| Reliable Deterioration | 14.3 | 8.7 | 1.63 [0.67, 4.15] | χ^2^ (1) = 2.81, *p* = .299 |
| Completed treatment | 68.6 | 57.5 | 1.61 [0.90, 2.89] | χ^2^ (1) = 4.85, *p* = .211 |
| Early termination (dropout) | 22.9 | 36.4 | 0.53 [0.28, 0.99] | χ^2^ (1) = 8.15, *p* = .115 |
|  | Mean (SE) | Mean (SE) | Cohen’s *d* |  |
| Number of sessions | 11.34 (0.95) | 8.84 (0.50) | 0.28 | *t*(367) = 2.34, *p* = .020 |
| Total session time (mins) | 521.71 (45.70) | 409.81 (29.13) | 0.22 | *t*(367) = 2.07, *p* = .040 |
| Number of cancelled or not attended sessions | 1.66 (0.35) | 2.13 (0.13) | 0.21 | *t*(367) = -1.29, *p* = .199 |
| Total duration of treatment (days) | 149.43 (15.38) | 145.85 (8.68) | 0.02 | *t*(367) = 0.20, *p* = .840 |
| PHQ-9 change | 6.83 (1.15) | 6.27 (0.34) | 0.09 | *t*(365) = 0.47, *p* = .641 |
| GAD-7 change | 5.86 (1.01) | 4.65 (0.31) | 0.21 | *t*(367) = 1.15, *p* = .252 |
| WSAS change | 6.14 (1.56) | 6.15 (0.53) | <0.01 | *t*(349) = -0.01, *p* = .998 |

*Notes.* All descriptives and statistical tests incorporated weighting to account for the matching ratio. Cohen’s *d* calculated using the pooled standard deviation.
